# Mobile device-based 3D scanning is superior to scoliometer in assessment of adolescent idiopathic scoliosis

**DOI:** 10.1007/s43390-024-01007-6

**Published:** 2024-12-12

**Authors:** Yousi Oquendo, Ian Hollyer, Clayton Maschhoff, Christian Calderon, Malcolm DeBaun, Joanna Langner, Nadine Javier, Xochitl Bryson, Ann Richey, Hiba Naz, Kali Tileston, Michael Gardner, John S. Vorhies

**Affiliations:** 1 Department of Orthopaedic Surgery, Hospital for Special Surgery, New York, NY USA; 2 Department of Orthopaedic Surgery, Stanford University School of Medicine, 453 Quarry Rd, 3rd Floor, MC 5658, Palo Alto, CA USA; 3 Department of Orthopaedic Surgery, Duke University School of Medicine, Durham, NC USA

**Keywords:** Scoliometer, 3D structure scanner, Scoliosis, Screening

## Abstract

**Purpose:**

Screening for adolescent idiopathic scoliosis (AIS) currently relies on clinical evaluations by trained practitioners, most commonly using a scoliometer. Modern structured light 3D scanning can generate high-quality 3D representations of surface anatomy using a mobile device. We hypothesized that a mobile-based 3D scanning system would provide accurate deformity assessments compared to a scoliometer.

**Methods:**

Between August 2020 and June 2022, patients 10–18 years being evaluated for AIS in our clinic with a scoliosis radiograph obtained within 30 days of clinic evaluation and no history of spinal surgery were enrolled. Patients had 3D scans taken in the upright and forward bend positions, and the largest angle of trunk rotation (ATR) was measured by a scoliometer. Image processing software was used to analyze trunk shift (TS), coronal balance (CB), and clavicle angle (CL) in the upright position and the largest ATR in the forward bend position. 3D and scoliometer measurements were correlated to major curve magnitude. Multiple logistic regression models were created based on 3D and scoliometer measurements, controlling for demographic covariates.

**Results:**

Two hundred and fifty-eight patients were included in this study. Mean coronal major curve magnitude was 25.7° (range 0–100), and 59% had a thoracic major curve. There were good-to-excellent correlations between 3D and radiographic measures of TS, CB, and CL (*r* = 0.79, rs = 0.80, and *r* = 0.64, respectively, *p* < 0.001). Correlations between 3D and radiographic measures of largest lumbar and thoracic ATR also demonstrated good correlations (*r* = 0.64 for both, *p* < 0.001). Using Akaike’s Information Criterion (AIC), a multivariable logistic regression model based on 3D scanning outperformed a scoliometer model.

**Conclusions:**

Mobile device-based 3D scanning of TS, CB, and TS identifies clinically relevant scoliotic deformity and is more predictive of radiographic curve magnitude than scoliometer in this population. This new modality may facilitate scoliosis screening by decreasing the need for trained personnel or dedicated equipment and clinical space to perform screening tests.

**Level of evidence:**

II.

## Introduction

Adolescent idiopathic scoliosis (AIS) is a structural multi-planar spinal deformity associated with vertebral rotation with an overall prevalence of 1–3% [1]. AIS is characterized by a coronal major curve magnitude of > 10° [2, 3]. Assessment of prognosis and treatment in pediatric patients primarily depends on skeletal maturity as well as the severity of the coronal major curve magnitude where curves < 20° are typically observed at regular intervals, curves between 20° and 45° are typically managed with bracing, and curves > 45° may benefit from operative management [4, 5]. Approximately 0.1% of curves fall in the operative range [6].

Early detection and effective nonoperative treatment can improve clinical outcomes and prevent curve progression to the surgical range. Therefore, the American Academy of Orthopaedic Surgeons, the Scoliosis Research Society, the Pediatric Orthopaedic Society of North America, and the American Academy of Pediatrics jointly support screening programs for earlier detection and management of AIS [7–11].

Current Scoliosis Research Society recommendations for AIS screening report that the best screening tool available for AIS uses the Adam’s forward bend test and a measurement of trunk rotation using a scoliometer [12, 13]. Scoliometers measure deviation from level across a patient’s back in the forward bending posture, which is recorded as the angle of trunk rotation (ATR). Studies have demonstrated a moderate correlation between scoliometer and radiographic coronal Cobb measurements (*r* = 0.572–0.685) [14].

Although scoliometers are relatively easy to use, there are inherent issues with the test, including variability of the placement of the device by practitioners and errors in positioning patients in the forward bend position [15]. Factors such as leg length discrepancy, tight hamstring muscles, or uneven leg positioning may result in an asymmetric or variable forward bending posture, resulting in a false positive or negative scoliometer screen [16]. Errors in modern screening methods have been a concern in the last few decades, as false positive referrals can lead to excessive costs [7, 17, 18]. In addition, patients who screen positive are often further evaluated by a full body-length anteroposterior and lateral radiograph [1, 19, 20]. Although current imaging techniques have reduced radiation exposure, false-positive referrals lead to unnecessary radiation, cost, and stress for patients and families [21–23].

The ideal AIS screening tool would have high accuracy, reproducibility, and sensitivity [24]. Given the limitations of current scoliometer screening, we sought to investigate an alternative screening test using relatively low-cost and radiation-free 3D modeling cameras attached to a mobile device [25]. This study aims to compare the reliability and validity of the 3D scanner for measuring trunk rotation in patients with AIS, and we hypothesize that a model based on the 3D scanning device will outperform the scoliometer in predicting patients with a major Curve magnitude > 20°.

## Methods

### Participants

After IRB approval, pediatric patients presenting to an outpatient pediatric orthopedic clinic affiliated with an academic referral center were prospectively recruited to the study between August 2020 and June 2022. All patients were between 10 and 18 years and were presenting for evaluation or follow-up of spinal deformity. Patients were included only if they had undergone an anteroposterior or posteroanterior scoliosis radiograph within 30 days of a clinic visit. Patients were excluded if they had a history of prior spine or chest surgery, a syndrome or neuromuscular condition associated with scoliosis, or a history of spinal fracture. Demographic data, including age, sex, race, ethnicity, and BMI, were recorded for each patient during their clinical encounter, and major curve magnitude was recorded for each visit. Radiographic measurements, including coronal major curve magnitude, trunk shift (TS), coronal balance (CB), and clavicle angle (CL), were made using Sectra radiographic imaging software (Sectra; Linköping, Sweden) by a trained research coordinator according to previously described methods [26].

### 3D scanning assessment

**Fig. 1:**
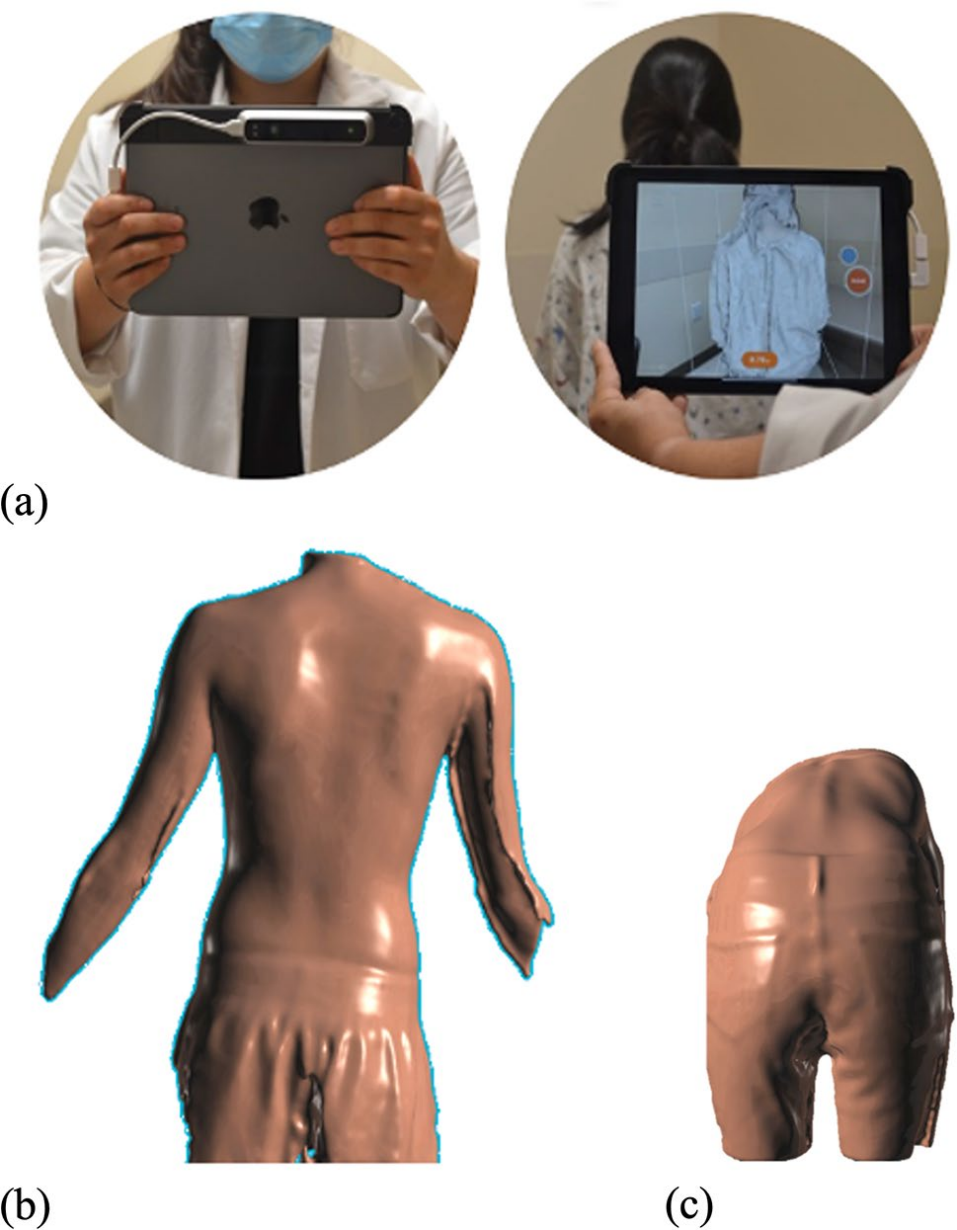
**a** Photo of the device used to collect 3D scanning data in the clinic and example of the screen display, as well as 3D reconstructions of patients in the **b** upright and **c** Adam’s forward bend position

3D measurements were obtained using Structure SensorTM Mark II device with Skanect software (Occipital Inc., Boulder, CO, USA), which uses structured light technology via an infrared laser scanner mounted onto an iPad Pro model MTXT2LL/A with iOS 14.2 (Apple, Cupertino, CA, USA) (Fig. 1). This scanner contains two infrared LEDs, an infrared structured light projector, and an infrared camera. The scanner projects an infrared grid reflected off objects and interpreted by the infrared camera. The software interprets variations in the position of the captured grid to determine the relative position of a captured image. The scanner has a resolution of 1280 × 960, a fish eye lens, a range of 4–10m, and a horizontal and vertical field of view of 59° and 46°, respectively [27]. During image collection, the operator moves in a 360° arc around the patient, collecting data with the scanner.

Once patients consented to the study, they were positioned with pants at their gluteal crease, hair tied with neck visible up to the hairline, and upper body undressed of loose-fitting clothing. The 3D images were obtained with the patient in two standardized positions. First, patients stood upright with their elbows fully extended and shoulders at 45° of abduction so as not to obstruct the lateral torso from the 3D scanner fields of view [28]. Next, patients were asked to bend over with arms hanging perpendicular to the ground, palms facing together, and knees extended with feet together in the position of Adam’s forward bend [29] (Fig. 1).

Measurements recorded by the 3D scanner included trunk shift (TS), coronal balance (CB), clavicle angle (CL), lumbar angle of trunk rotation (LATR), thoracic angle of trunk rotation (TATR), and largest angle of trunk rotation, which was based on techniques from the spine deformity study group manual [29]. Solidworks Student Edition 2020 (Dassault Systèmes; Vélizy-Villacoublay, France) was used to measure TS and CB from 3D scans.

### Scoliometer assessment

After each patient had completed the 3D image collection, a trained research coordinator measured the maximum thoracic and lumbar angle of trunk rotation angle using a scoliometer (Mizuho, USA) [29]. Patients again assumed the position of Adam’s forward bend test. At the same time, the measurer stood behind the patient and assessed the horizontal plane of the spine, placing the half-circle cutout of the scoliometer over the top of the spinous process and measuring the maximal ATR on the thoracic and lumbar spine. The height of the patient’s bending position was adjusted as needed so that the deformity of the spine was most pronounced to find the maximum thoracic or lumbar ATR.

### Statistical analysis

Baseline correlations among upright X-ray vs. 3D scan measures, bending scoliometer vs. 3D scan measures, and all measures vs. curve magnitudes were analyzed using Pearson’s and Spearman’s correlation tests. A power analysis was also performed and indicated that to detect a correlation of 0.20 or above with 80% power, the study would require 193 participants. Multivariable logistic regression models were generated to evaluate the ability of the scoliometer and 3D measurements to predict major curve magnitude > 20°. These models were constructed using BMI with or without adjustments for age and sex, as these predictor variables are likely to be readily available to practitioners who may screen for scoliosis. We chose a major curve magnitude cutoff of > 20° based on previous literature and expert opinion, as treatment is not typically offered to patients with curves < 20° [30]. Akaike’s Information Criterion (AIC) and McFadden’s pseudo-*R*^2^ values were used to determine optimal models, where models with lower AIC values (no range) and higher pseudo-*R*^2^ (range 0–1) are indicative of a better predictive model. AIC analysis is widely used to compare different models using the same data and has been previously utilized to evaluate diagnostic tests in multiple clinical fields [31–37]. AIC values were calculated for all combinations between each cutoff point of the LATR measured by scoliometer and the chance of having a major curve magnitude > 20°. AIC and pseudo-*R*^2^ values were then calculated for all combinations between each cutoff point of the 3D variables and the odds of having a major curve magnitude > 20°. All analyses were performed by a trained statistician in RStudio version 2023.03.1+446 (Boston, MA) using a two-sided significance level of 0.05.

## Results

### Demographics and radiographic measurements

A total of 258 patients were included in this study (Table 1). The mean age at visit was 13.6 ± 2.0 years (10–17 years), and 188 (73%) were female. Of the female participants, 39 (22%) were premenarchal. About half of patients (45%) were Risser stage 2 or lower and had no bracing history (164, 64%) at initial presentation. All Risser stages were represented in the sample, and most patients were White/Caucasian (126, 48%).

**Table 1:** Demographic data

Age (mean, range)	13.6	10–18
BMI percentile (mean, SD)^a^	50.9	27.1
Sex (*n*, %)		
Female	188	73%
Male	70	27%
Risser (*n*, %)^b^		
0	65	28%
1	25	11%
2	14	6%
3	18	8%
4	91	39%
5	20	9%
History of bracing (*n*, %)		
No	164	64%
Yes	91	36%
Menarche status (*n*, %)		
Post	142	78%
Pre	39	22%

^a^Based on CDC growth charts

^b^In some patents the pelvis was cropped so assessment was not possible

**Fig. 2:**
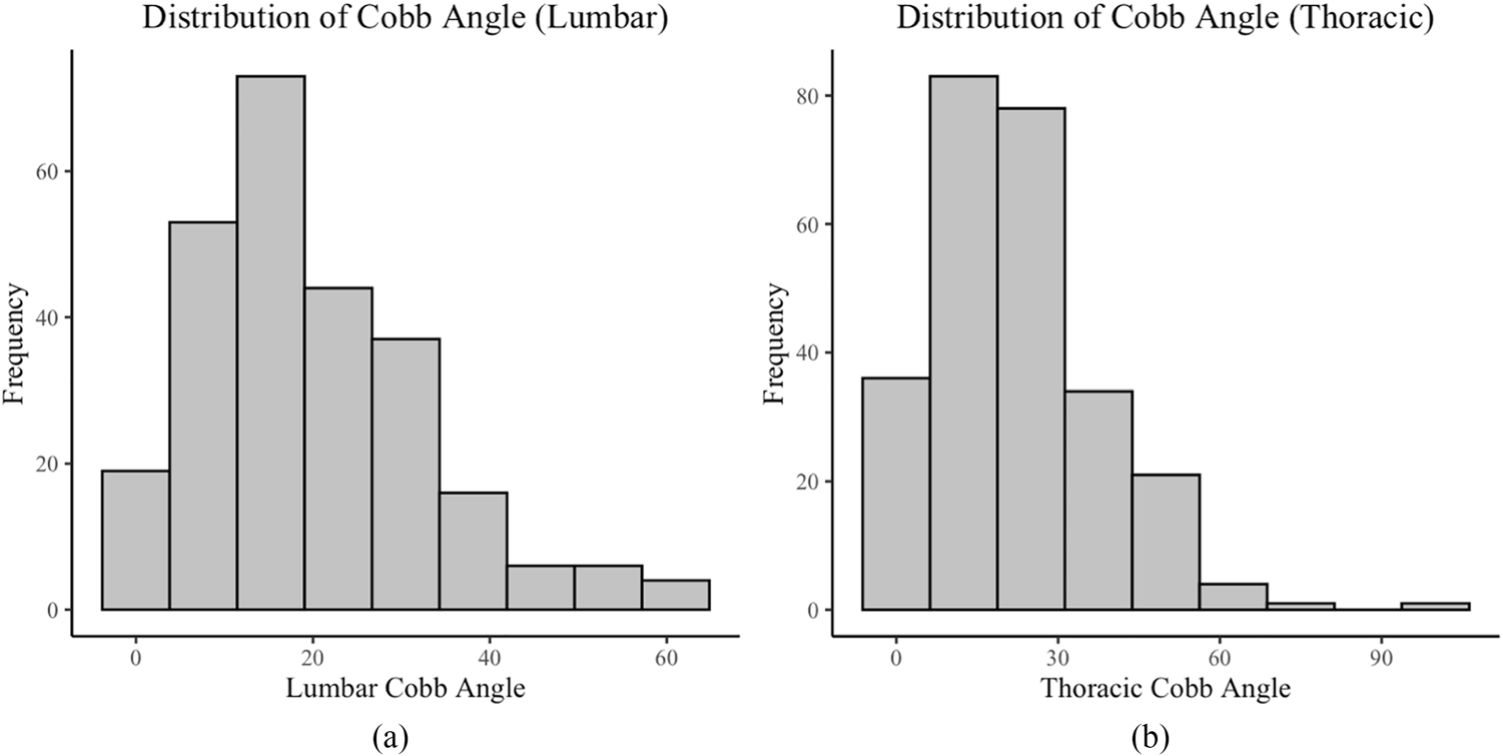
Distribution of **a** lumbar and **b** thoracic major curves included in the final analysis. Within these groups, 48% of patients with a major thoracic and 55% of patients with a major lumbar curve had curve magnitudes < 20°

Mean major curve magnitude was 25.7° (range 0–100), and 59% had a thoracic major curve (Table 2). Patients had an average lumbar curve of 19.8° [range 0–61, interquartile range (IQR) 10–28], an average thoracic curve of 22.1° (range 0–100, IQR 11–31), an average trunk shift of 10.4 mm (range 0–64, IQR 4–14), coronal balance of 12.4 mm (range 0–57, IQR 6–19), and a clavicle angle of 1.9° (range 0–8, IQR 1–3). Within the cohort, 48% of patients had a thoracic coronal curve < 20°, while 55% had a lumbar coronal curve < 20° (Fig. 2).

**Table 2:** Radiographic parameters

Major curve magnitude (mean, range)	25.7	(0–100)
Thoracic major curve (*n*, %)	152	59%
Lumbar Cobb angle (mean, range)	19.8	(0—61)
Thoracic Cobb angle (mean, range)	22.1	(0–100)
Trunk Shift angle (mean, range)	10.4	(0–64)
Coronal balance angle (mean, range)	12.4	(0–57)
Clavicle angle (mean, range)	1.9	(0–8)

### Correlations between upright radiographic and 3D measures of coronal balance

**Fig. 3:**
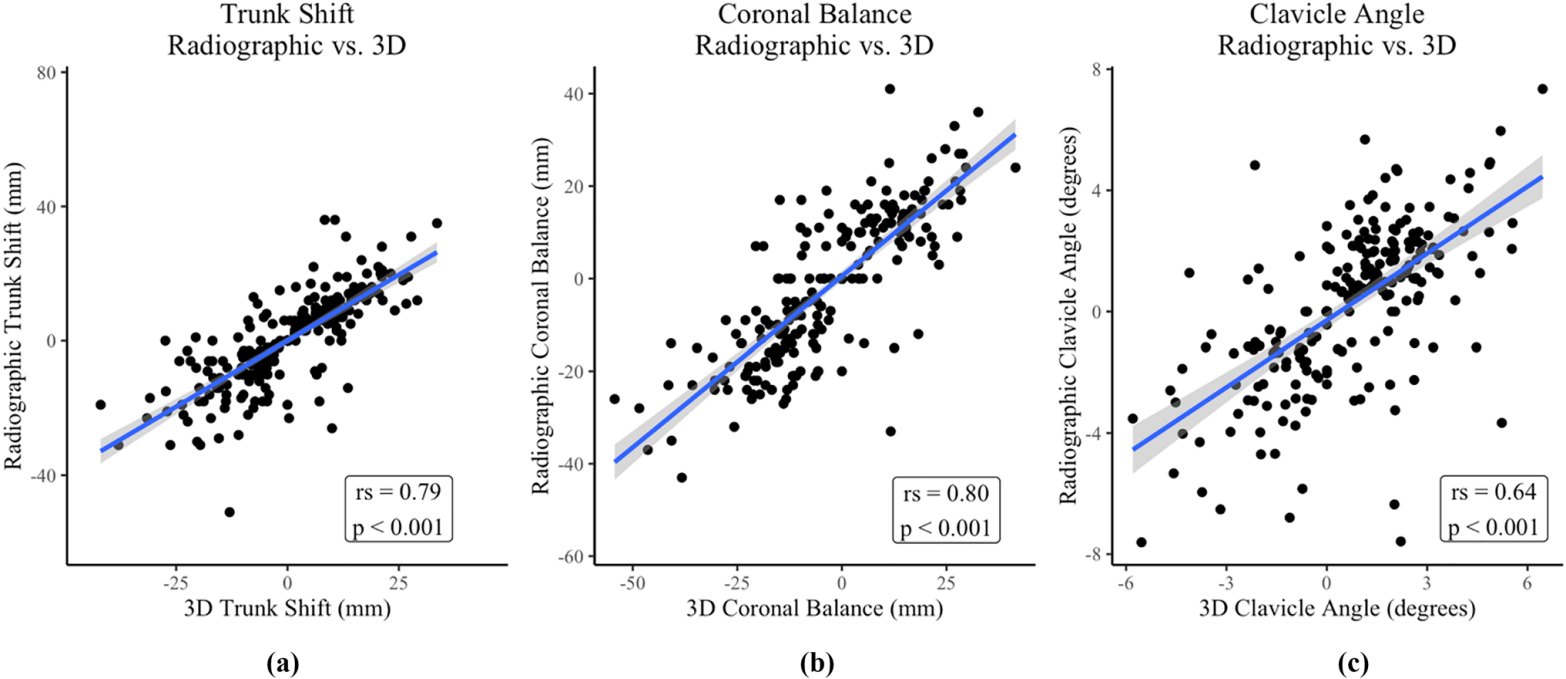
Correlation of radiographic parameters to 3D scanner. **a** Radiographic vs. 3D assessment of trunk shift. **b** Radiographic coronal balance vs. 3D coronal balance. **c** Radiographic clavicle angle vs. 3D clavicle angle

Analysis of the correlations between radiographic and 3D measures of upright coronal balance demonstrated good-to-excellent relationships (Table 3). Radiographic and 3D measures of trunk shift and coronal balance yielded a Spearman’s correlation of *r* = 0.79 and *r*_s_ = 0.80, respectively, while the correlation between radiographic and 3D clavicle angle was *r* = 0.64 (*p* < 0.001 for all) (Table 3, Fig. 3).

**Table 3:** Correlations between test variables

Measurement correlations: X-ray vs. 3D (upright)	*r*	*p* value
Trunk shift	0.79	< 0.001*
Coronal balance	0.80	< 0.001*
Clavicle angle	0.64	< 0.001*

Measurement correlations: scoliometer vs. Cobb angle	*r*_*s*_	*p* value
Lumbar angle of trunk rotation	0.35	< 0.001*
Thoracic angle of trunk rotation	0.44	< 0.001*

Measurement correlations: 3D vs. Cobb angle	*r*_*s*_	*p* value
Lumbar angle of trunk rotation	0.64	< 0.001*
Thoracic angle of trunk rotation	0.64	< 0.001*

*r* = Pearson’s correlation coefficient; *r*_*s*_ = Spearman’s correlation coefficient

*Statistically significant

### Correlations between scoliometer and 3D measurements vs. radiographic coronal major curve

**Fig. 4:**
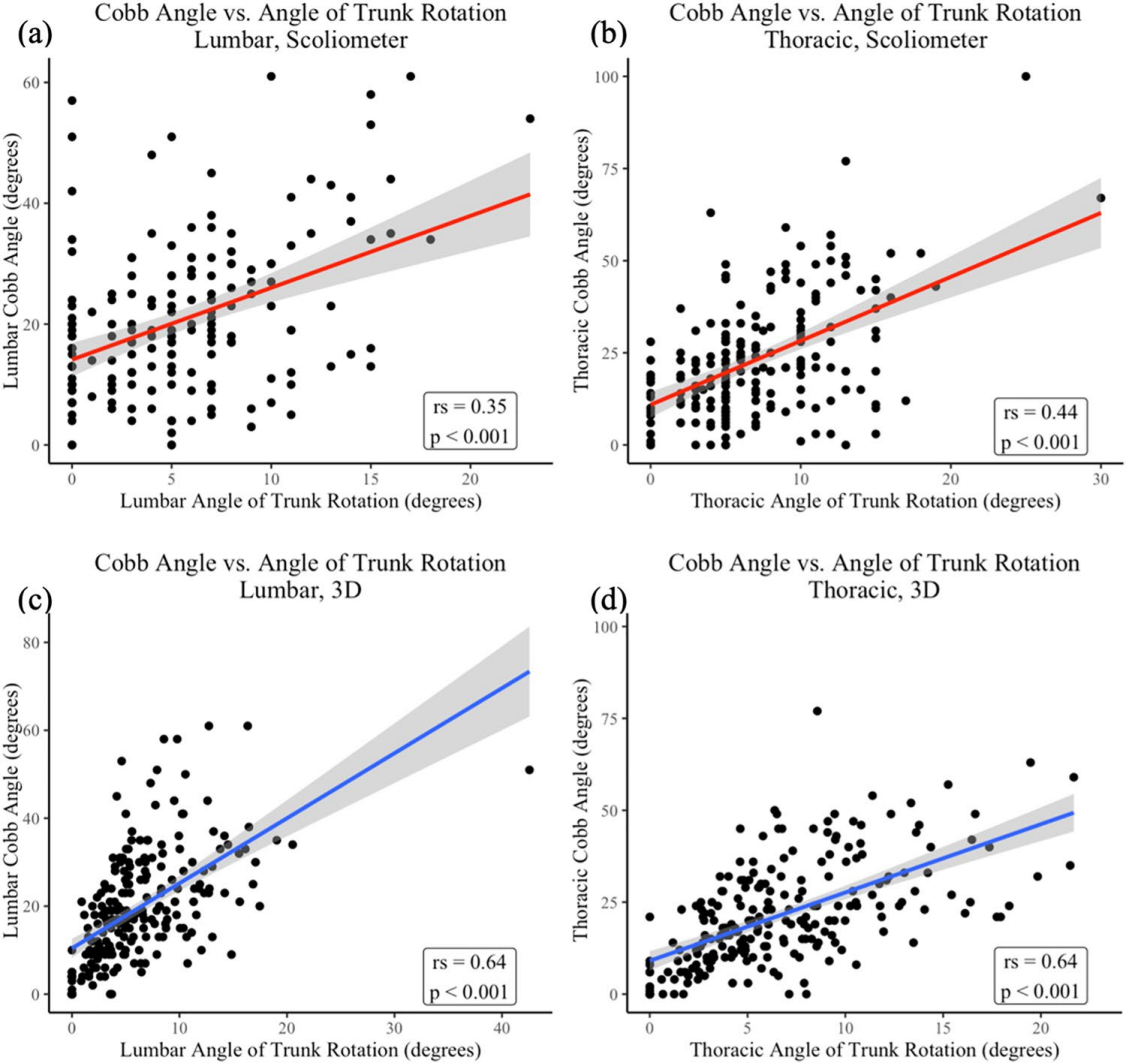
Correlations of scoliometer and 3D scanner measurements with radiographic coronal major curves. **a** Scoliometer measurement of largest lumbar ATR vs. lumbar major Cobb angle. **b** Scoliometer measurement of largest thoracic ATR vs. thoracic major Cobb angle. **c** 3D measurement of largest lumbar ATR vs. lumbar major Cobb angle. **d** 3D measurement of largest thoracic ATR vs. thoracic major Cobb angle

Relationships between scoliometer measurements of the largest lumbar ATR and thoracic ATR with radiographic major curve magnitude demonstrated a weak (*r*_s_ = 0.35, *p* < 0.001) and moderate (*r*_s_ = 0.44, *p* < 0.001) correlation, respectively. Comparing the 3D assessment of the largest lumbar ATR and thoracic ATR with radiographic major curve magnitude, each demonstrated strong correlations (*r* = 0.64, *p* < 0.001 for both) (Table 3, Fig. 4).

### Multivariable logistic regression modeling to predict Cobb > 20°

Parallel multivariable logistic regression models were created using largest scoliometer measurements or all 3D measurements as the predictors of major curve magnitude > 20° (Table 4). We generated a model controlling for BMI, given that body habitus may affect the accuracy with which surface topography reflects underlying skeletal structure. The scoliometer + BMI model had an AIC = 242.38 and pseudo-*R*^2^ = 0.33. The 3D measurement + BMI model had an AIC = 207.64, pseudo-*R*^2^ = 0.44. The 3D measurement model outperformed the scoliometer model, with lower AIC and higher pseudo R^2^ values.

**Table 4:** Multivariable logistic regression models

Model performance	AIC	Pseudo *R*^2^
Scoliometer (BMI only regression)	242.38	0.33
3D measurements (BMI only regression)	207.64	0.44
Scoliometer (BMI, age, sex regression)	211.77	0.27
3D measurements (BMI, age, sex regression)	200.12	0.33

We also generated parallel scoliometer and 3D-based multivariable logistic regression models to control for BMI, patient age, and sex in an attempt to simulate the information available to a provider screening for scoliosis (Table 4). The scoliometer model had an AIC = 211.77 and pseudo R^*2*^ = 0.27. The 3D measurement model had an AIC = 200.12 and pseudo R^*2*^ = 0.33. The 3D measurement model again outperformed the scoliometer model.

## Discussion

This study aimed to evaluate the ability of measurements derived from a mobile device-based 3D scanner to predict the presence of a scoliotic curve > 20° and compare that to the current standard screening methodology using a scoliometer. In the last century, multiple screening methods have been developed to detect AIS, including Adam's forward bend test, scoliometer, ultrasound, and back surface topography [15, 38–42]. However, these methods require a trained practitioner to perform the test and interpret results or stationary specialized equipment assembled beforehand in a clinical space [15, 38, 40–42]. Mobile device-based 3D methods do not require any specialized clinical room setup and can be performed in many settings. 3D scanning based on structured light can create noninvasive elaborations of 3D meshes of the anatomic surfaces of a patient's back in the upright and Adam's forward bend position. In contrast, a scoliometer only assesses focal areas of truncal asymmetry [25, 43, 44].

This study found that 3D measures of trunk shift, coronal balance, and clavicle angle had a good-to-excellent correlation with their radiographic counterpart measurements. Furthermore, surface topography-based measurement of ATR had a better correlation with radiographic major curve magnitude than scoliometer-based ATR measurement. The multiple regression model incorporating 3D scanning measures of trunk shift, coronal balance, clavicle angle, and most significant angle of trunk rotation, controlling for age, sex, and BMI, demonstrated superior test characteristics compared to the multiple regression model using scoliometer-based measurement of trunk rotation.

Prior work has shown that 17–42% and 40–67% of all referrals for AIS to an orthopedic specialist have major curve magnitudes < 10° and < 20°, respectively [7, 30, 45]. Decreasing referrals for children with < 20° curves has many potential benefits, such as reducing unnecessary stress, decreasing healthcare burden, and lowering cost, as research has shown the average scoliosis referral costs $780 per visit [30]. Initial evaluation and subsequent monitoring of spinal deformity in children also often involves multiple radiographic assessments, which can have potentially adverse consequences for some patients, such as a long-term higher chance of mortality from breast cancer [2, 46–48].

One of the drawbacks to the 3D scanning model described here is the cost. Scoliometers cost around $100, while the *Structure SensorTM Mark II* costs around $400 and requires additional devices, such as an iPad, to interface with the device [49]. Although decreasing unnecessary referrals would save patients and the healthcare system money, the investment cost for the 3D scanning technology may be a barrier to community screening programs in schools or primary care clinics. Additionally, while the image acquisition process is simple, performing all 3D scanning measurements is labor intensive and may require more work to scale in its current form.

The strength of this study was the large sample size used to generate a direct comparative model between the two modes of scoliosis screening: a scoliometer vs. a mobile device-based 3D scanner. The limitation of this study is that the cohort of patients included was studied in a single referral center and geographic region, which reduces the generalizability of the results. We evaluated a convenient sample of patients with radiographs taken from a pediatric orthopedic clinic. This may have resulted in a sample biased toward patients with more trunk deformity than the general population. Though our study populations did include many patients who did not meet the radiographic definition of scoliosis, all were referred for specialist evaluation based on suspicion of spinal deformity by their primary care providers, perhaps due to trunk or chest wall asymmetry, even in the absence of scoliosis. Further research is needed to compare the performance of this 3D-based screening tool with scoliometer screening in the general population.

## Conclusion

Here, we demonstrate the ability of mobile device-based 3D scanning to quantify clinically significant trunk asymmetry. We show that 3D topographical measurements correlate with their radiographic counterparts. Our results indicate that a 3D measurement-based prediction model was more effective at predicting the presence of scoliosis than a model based on the angle of trunk rotation evaluated with a scoliometer. Further research is needed to determine if this method is generalizable to other patient populations.

## Data Availability

The data that support the findings of this study are available from the corresponding author, JSV, upon request.
