# Correction: Mobile device-based 3D scanning is superior to scoliometer in assessment of adolescent idiopathic scoliosis

**DOI:** 10.1007/s43390-025-01046-7

**Published:** 2025-01-23

**Authors:** Yousi Oquendo, Ian Hollyer, Clayton Maschhoff, Christian Calderon, Malcolm DeBaun, Joanna Langner, Nadine Javier, Xochitl Bryson, Ann Richey, Hiba Naz, Kali Tileston, Michael Gardner, John S. Vorhies

**Affiliations:** 1 Department of Orthopaedic Surgery, Hospital for Special Surgery, New York, NY USA; 2 Department of Orthopaedic Surgery, Stanford University School of Medicine, 453 Quarry Rd, 3rd Floor, MC 5658, Palo Alto, CA USA; 3 Department of Orthopaedic Surgery, Duke University School of Medicine, Durham, NC USA

**Correction: Spine Deformity** 10.1007/s43390-024-01007-6

The article **Mobile device-based 3D scanning is superior to scoliometer in assessment of adolescent idiopathic scoliosis**, written by Yousi Oquendo, Ian Hollyer, Clayton Maschhoff, Christian Calderon, Malcolm DeBaun, Joanna Langner, Nadine Javier, Xochitl Bryson, Ann Richey, Hiba Naz, Kali Tileston, Michael Gardner and John S. Vorhies, was originally published under exclusive license to The Author(s), under exclusive licence to Scoliosis Research Society 2024. As a result of the subsequent decision to publish the article under the open access model, the article’s copyright notice was changed on December 17, 2024 to © The Authors 2024 and the article is now distributed under a Creative Commons Attribution 4.0 International License, which permits use, sharing, adaptation, distribution and reproduction in any medium or format, as long as you give appropriate credit to the original author(s) and the source, provide a link to the Creative Commons licence, and indicate if changes were made.

The images or other third party material in this article are included in the article’s Creative Commons licence, unless indicated otherwise in a credit line to the material. If material is not included in the article’s Creative Commons licence and your intended use is not permitted by statutory regulation or exceeds the permitted use, you will need to obtain permission directly from the copyright holder.

To view a copy of this licence, visit http://creativecommons.org/licenses/by/4.0/.

